# The PUB4 E3 Ubiquitin Ligase Is Responsible for the Variegated Phenotype Observed upon Alteration of Chloroplast Protein Homeostasis in Arabidopsis Cotyledons

**DOI:** 10.3390/genes12091387

**Published:** 2021-09-06

**Authors:** Nicolaj Jeran, Lisa Rotasperti, Giorgia Frabetti, Anna Calabritto, Paolo Pesaresi, Luca Tadini

**Affiliations:** Dipartimento di Bioscienze, Università degli Studi di Milano, 20133 Milano, Italy; nicolaj.jeran@unimi.it (N.J.); lisa.rotasperti@unimi.it (L.R.); giorgia.frabetti@studenti.unimi.it (G.F.); anna.calabritto@studenti.unimi.it (A.C.); paolo.pesaresi@unimi.it (P.P.)

**Keywords:** chloroplast, ubiquitination, variegated phenotype, protein homeostasis

## Abstract

During a plant’s life cycle, plastids undergo several modifications, from undifferentiated pro-plastids to either photosynthetically-active chloroplasts, ezioplasts, chromoplasts or storage organelles, such as amyloplasts, elaioplasts and proteinoplasts. Plastid proteome rearrangements and protein homeostasis, together with intracellular communication pathways, are key factors for correct plastid differentiation and functioning. When plastid development is affected, aberrant organelles are degraded and recycled in a process that involves plastid protein ubiquitination. In this study, we have analysed the Arabidopsis *gun1-102 ftsh5-3* double mutant, lacking both the plastid-located protein GUN1 (Genomes Uncoupled 1), involved in plastid-to-nucleus communication, and the chloroplast-located *FTSH*5 (Filamentous temperature-sensitive H5), a metalloprotease with a role in photosystem repair and chloroplast biogenesis. *gun1-102 ftsh5-3* seedlings show variegated cotyledons and true leaves that we attempted to suppress by introgressing second-site mutations in genes involved in: (i) plastid translation, (ii) plastid folding/import and (iii) cytosolic protein ubiquitination. Different phenotypic effects, ranging from seedling-lethality to partial or complete suppression of the variegated phenotype, were observed in the corresponding triple mutants. Our findings indicate that Plant U-Box 4 (PUB4) E3 ubiquitin ligase plays a major role in the target degradation of damaged chloroplasts and is the main contributor to the variegated phenotype observed in *gun1-102 ftsh5-3* seedlings.

## 1. Introduction

Albinotic and variegated mutants have been widely used to investigate chloroplast biogenesis and the communication pathways between the nuclear-cytosolic compartment and chloroplasts [1,2,3]. Among these, Arabidopsis *ftsh5* (*var1*) and *ftsh2* (*var2*) mutants, devoid of two subunits of the thylakoid transmembrane metalloprotease complex *FTSH* (Filamentation Temperature Sensitive protein H), responsible for photosystem II (PSII) biogenesis and repair, and characterized by a leaf variegated phenotype, contributed greatly in revealing the strict link between chloroplast protein homeostasis and proper chloroplast biogenesis [4,5,6,7,8]. Transmission electron microscopy analyses of *ftsh2* and *ftsh5* leaves revealed, indeed, correctly-shaped chloroplasts in the green sectors, while the white sectors showed miss-shaped plastids with highly vacuolated organelle structures [6,9]. Furthermore, second-site suppressor screens aimed to identify mutations able to suppress the *ftsh* leaf variegated phenotype, so-called Suppressors of Variegation (SVR), allowed the identification of several nuclear genes encoding plastid-located proteins mostly involved in rRNA maturation, translation, protein folding and protein degradation [3,10,11,12,13,14]. Overall, these findings indicated that the differentiation of functional chloroplasts requires an optimal balance between the rate of plastid protein synthesis and the activity of the plastid protein quality control machinery [15].

Recently, the variegated phenotype was also observed in cotyledons of *ftsh2* and *ftsh5* seedlings upon introgression of the *gun1* knock-out mutation (for further details see Tadini et al. [16]). GUN1 is a chloroplast-localized pentatricopeptide repeat (PPR) protein required during the early stages of chloroplast biogenesis and upon alterations of plastid gene expression and chloroplast protein homeostasis [16,17,18,19,20,21]. In particular, *gun1 ftsh2* cotyledons are characterized by the presence of highly vacuolated plastids, without any traces of thylakoid membranes, while *gun1 ftsh5* cotyledons show a less severe albino-variegated phenotype and possess cells with either functional chloroplasts or plastids with budding vesicles, an evidence of the ongoing plastid degradation process [16].

Recent studies have highlighted that the ubiquitin-dependent modification of proteins located on the chloroplast outer membrane is a pivotal event, at the basis of chloroplast adaptation to stress conditions, involving either targeted protein removal through the ubiquitin–proteasome system, or selective, whole-chloroplast degradation, based on the severity of the chloroplast damages [22,23,24]. Suppressor of PPI1 locus1 (SP1) and Plant U-Box 4 (PUB4) are the two main E3 ubiquitin ligases reported to be involved in chloroplast ubiquitination, so far [22,24,25]. SP1 is embedded in the outer envelope of the chloroplast and, by ubiquitination of Translocon of Outer membrane of Chloroplast (TOC) components, confers the ability to isolate the chloroplasts from the bulk of plastid precursor proteins in the cytoplasm, thus modulating the quantity and the quality of the imported pre-proteins [22,23,26]. On the other hand, PUB4 is soluble in the cytoplasm and acts on still unidentified proteins located on the chloroplast outer envelope, serving to target damaged chloroplasts for degradation in response to ROS stress. This would provide a chloroplast quality control mechanism to reduce the risk of further ROS accumulation [24,27].

In this work we attempted to suppress the variegated phenotype of *gun1-102 ftsh5-3* cotyledons through the introgression of additional mutations in nuclear genes, encoding proteins with roles in: (i) plastid translation, (ii) plastid folding/import and (iii) cytosolic protein ubiquitination. We demonstrated that the introgression of *pub4-7* mutation into the *gun1-102 ftsh5-3* genetic background suppresses the variegated phenotype of *gun1-102 ftsh5-3* cotyledons. In particular, the degenerating *gun1-102 ftsh5-3* plastids are replaced by functional chloroplasts in *gun1-102 ftsh5-3 pub4-7* cotyledons, indicating that the PUB4-dependendent chloroplast quality control mechanism is active in *gun1-102 ftsh5-3* cotyledons and is at the basis of the variegated phenotype.

## 2. Materials and Methods

### 2.1. Plant Material and Growth Conditions

Arabidopsis (*Arabidopsis thaliana*, genetic background Col-0) wild-type and mutant seeds were grown on soil in a climate chamber under long-day conditions (16 h at 100 μmol photons m^−2^ s^−1^ light and 8 h dark, at 22 °C temperature). Genetic loci and T-DNA flanking regions of insertional mutant lines used in this work are described in Figure S1. Primer sequences for genotype determination are listed in Table S1. Multiple mutants were generated by manual crossing and identified by PCR-based segregation analyses of F2 populations, with the only exception being the *gun1-102 ftsh5-3 pub4-7* mutant, where the *pub4-7* mutant allele was generated using the CRISPR-Cas9 genome editing strategy. In particular, the *gun1-102 ftsh5-3 pub4-7* triple mutant was generated by targeting the fourth exon of the *PUB4* locus in the *gun1-102 ftsh5-3* mutant background using the pHEE401E vector described by Xing et al. [28]. Mutant plants carrying the mutation of interest and devoid of the Cas9 endonuclease were selected in the T3 generation. Primer sequences used for guide RNA design are listed in Table S1. Cotyledon and leaf area were determined by the ImageJ software (http://imagej.nih.gov/ij/index.html, accessed on 15 August 2021). The Variegation Index (V.I.) was calculated as the ratio of green area over the total area of the organ.

### 2.2. Chlorophyll Fluorescence Measurements and Chlorophyll Quantification

The imaging Chl fluorometer (Walz Imaging PAM; https://walz.com/, accessed on 15 August 2021) was used to measure in vivo Chl a fluorescence. Six plants of each genotype were analyzed at 6 and 12 days after sowing (DAS) and average values plus-minus standard deviations were calculated. Dark-adapted plants were exposed to blue measuring beam (intensity 4) and a saturating light flash (intensity 4) to obtain the maximum quantum yield of PSII, *Fv*/*Fm*. A 5-min exposure to actinic light (36 μmol photons m^−2^ s^−1^) allowed for the calculation of the effective quantum yield of PSII, Y_II_. For Chl quantification, 100 mg (fresh weight) of 6 DAS seedlings were ground in liquid nitrogen and extracted in 90% acetone. Chlorophyll (Chl) a and b concentrations were measured according to Porra et al. [29]. Measurements were performed in triplicate.

### 2.3. Transmission Electron Microscopy (TEM) Analyses

For TEM observations, tissue samples from fully expanded cotyledons of Col-0, *ftsh5-3*, *pub4-2* and *gun1-102 ftsh5-3 pub4-7* seedlings were prepared according to Tadini et al. [16]. In particular, 6 DAS seedlings were fixed in 3.3% (*v*/*v*) paraformaldehyde and 1.2% (*v*/*v*) glutaraldehyde in 0.1 M phosphate buffer (pH 7.4) at 4 °C for 2 h and post-fixed in 1% OsO_4_ in the same buffer for 2 h. Samples were then dehydrated in an ethanol series and embedded in Spurr’s resin. Ultrathin sections were stained with 2% uranyl acetate and lead citrate and observed with a Jeol 100SX TEM (Jeol; https://www.jeol.co.jp/, accessed on 15 August 2021) operating at 80 KV.

### 2.4. Quantitative Real-Time PCR (qRT-PCR) Analyses

Total RNA was isolated from 6 DAS Col-0 and mutant seedlings. For qRT-PCR analyses, 1 µg of total RNA was treated with iScript™ gDNA Clear cDNA Synthesis Kit (Bio-Rad; https://www.bio-rad.com/, accessed on 15 August 2021) for genomic DNA digestion and first-strand cDNA synthesis. qRT-PCR analyses were performed on a CFX96 Real-Time system (Bio-Rad; https://www.bio-rad.com/, accessed on 15 August 2021) using primer pairs listed in Table S1. *PP2AA3* (*AT1G13320*) transcripts were used as internal reference, as described [30]. Data obtained from three biological and three technical replicates for each sample were analyzed with the Bio-Rad CFX Maestro 1.1 (v 4.1) (Bio-Rad; https://www.bio-rad.com/, accessed on 15 August 2021).

### 2.5. Protein Sample Preparation and Immunoblot Analyses

For immunoblot analyses, cotyledons from 6 DAS seedlings were homogenized in Laemmli sample buffer (20% [*v*/*v*] glycerol, 4% [*w*/*v*] SDS, 160 mM Tris-HCl pH 6.8, 10% [*v*/*v*] 2-mercaptoethanol) to a final concentration of 0.1 mg µL^−1^ (fresh weight/Laemmli sample buffer). Samples were incubated at 65 °C for 15 min and, after a centrifugation step at 16,000× *g* for 10 min, the supernatant was incubated at 95 °C for 5 min. Protein extracts corresponding to 4 mg (fresh-weight) seedlings were loaded onto SDS–PAGE (10% [*w*/*v*] acrylamide) gels and transferred to polyvinylidene-difluoride (PVDF) filters (0.20 µm pore size). Replicate membranes were immuno-decorated with specific antibodies.

Intact chloroplasts were isolated from 100 mg (fresh weight) 6 DAS seedlings according to Kunst [31], with a few changes. Samples were homogenized in 1 mL of 45 mM sorbitol, 20 mM Tricine-KOH pH 8.4, 10 mM EDTA, 10 mM NaHCO_3_ and 0.1% (*w*/*v*) BSA fraction V, supplemented with proteinase inhibitor cocktail (cOmplete™, COEDTAF-RO, Roche; https://www.roche.com/, accessed on 15 August 2021), and centrifuged for 7 min at 700× *g*. The supernatant was discarded, while the pellet was washed twice with 1 mL of homogenization buffer. After a centrifugation step (7 min at 700× *g*), the pellet was collected as the intact chloroplast fraction. The level of signals was quantified by the ImageJ software (http://imagej.nih.gov/ij/index.html, accessed on 15 August 2021).

Antibodies specific for LHCB5 (AS01 009), VIPP1 (AS06 145), CLPB3 (AS09 459), cpHSC70-1 (AS08 348), CPN60 (AS12 2613), HSP90-1 (AS08 346) and UBQ11 (AS08 307A) were obtained from Agrisera (https://www.agrisera.com/, accessed on 15 August 2021). The HSC70-4 antibody was purchased from Antibodies-online (https://www.antibodies-online.com/, accessed on 15 August 2021).

### 2.6. Accession Numbers

The Arabidopsis Genome Initiative accession numbers for the genes mentioned in this work can be found at TAIR (https://www.arabidopsis.org/, accessed on 15 August 2021): *GUN1* (*AT2G31400*), *FTSH5* (*AT5G42270*), *FUG1* (*AT1G17220*), *PRPS21* (*AT3G27160*), *cpHSC70-1* (*AT4G24280*), *SP1* (*AT1G63900*), *PUB4* (*AT2G23140*), *PP2AA3* (*AT1G13320*) and *HSFA2* (*AT2G26150*).

## 3. Results

### 3.1. The Cytosolic E3 Ubiquitin Ligase PUB4 Is Responsible for the Variegated Phenotype Observed in Cotyledons and Leaves of gun1-102 ftsh5-3 Seedlings

**Figure 1:**
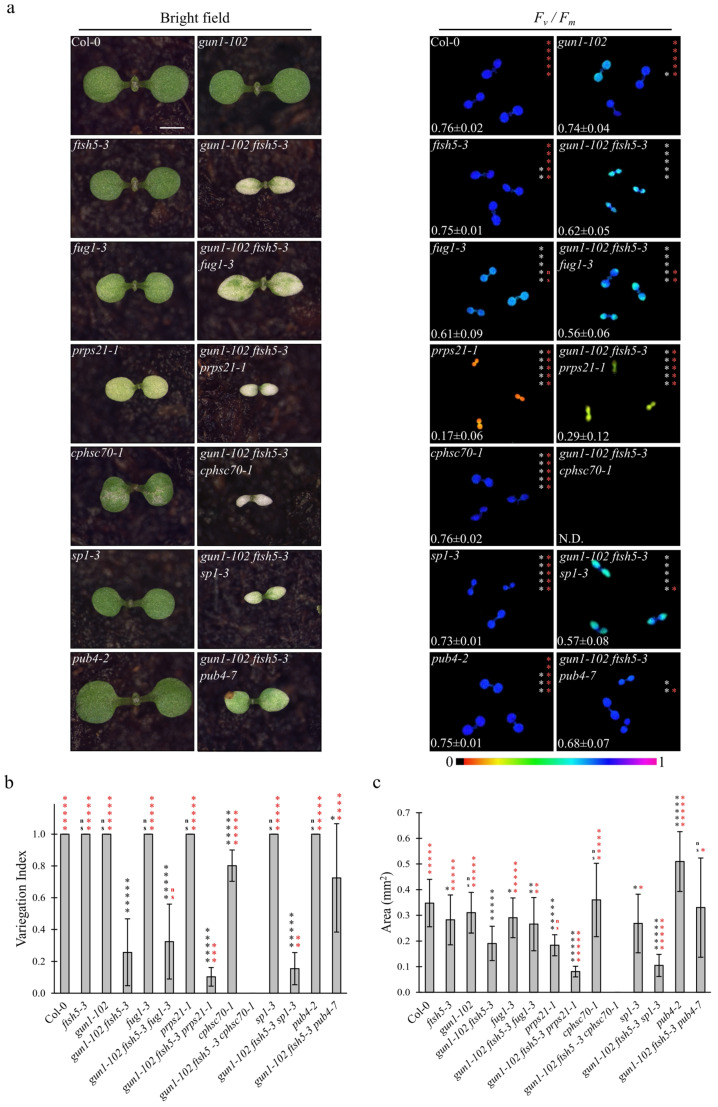
Visible phenotypic characteristics and photosynthetic performance of Arabidopsis Col-0 and mutant seedlings at cotyledon stage. (**a**) Visible phenotypes and maximum quantum yield of PSII (*F_v_*/*F_m_*) of Col-0, *gun1-102*, *ftsh5-3*, *gun1-102 ftsh5-3*, *fug1-3*, *gun1-102 ftsh5-3 fug1-3*, *prps21-1*, *gun1-102 ftsh5-3 prps21-1*, *cphsc70-1*, *gun1-102 ftsh5-3 cphsc70-1*, *sp1-3*, *gun1-102 ftsh5-3 sp1-3*, *pub4-2*, *gun1-102 ftsh5-3 pub4-7* cotyledons at six days after sowing (DAS) grown on soil. Scale bar corresponds to 1 mm. *F_v_*/*F_m_* parameter was measured through the IMAGING PAM Fluorimeter (Walz) and reported both in false colour (black equals to 0, violet to 1) and as average ± standard deviation of six independent measurements; N.D.: not detected. Asterisks indicate statistical significance with respect to Col-0 (white) or *gun1-102 ftsh5-3* (red) as evaluated by Student’s *t*-test and Welch correction (* *p* < 0.05; ** *p* < 0.01; *** *p* < 0.001; **** *p* < 0.0001; ***** *p* < 0.00001; ns: not significant). (**b**) Average Variegation Index (V.I.) calculated as ratio between the green area over the total area of the cotyledon of Col-0 and mutant seedlings grown on soil at 6 DAS. Error bars indicate standard deviations of at least six independent measurements. Asterisks indicate statistical significance with respect to Col-0 (black) or gun1-102 ftsh5-3 (red) as evaluated by Student’s t-test and Welch correction (* *p* < 0.05; ** *p* < 0.01; *** *p* < 0.001; **** *p* < 0.0001; ***** *p* < 0.00001; ns: not significant). (**c**) Average area in mm^2^ of single cotyledons of the indicated genotypes grown on soil at 6 DAS. Error bars indicate standard deviations of at least six independent measurements. Asterisks indicate statistical significance with respect to Col-0 (black) or *gun1-102 ftsh5-3* (red) as evaluated by Student’s *t*-test and Welch correction (* *p* < 0.05; ** *p* < 0.01; *** *p* < 0.001; **** *p* < 0.0001; ***** *p* < 0.00001; ns: not significant).

**Figure 2:**
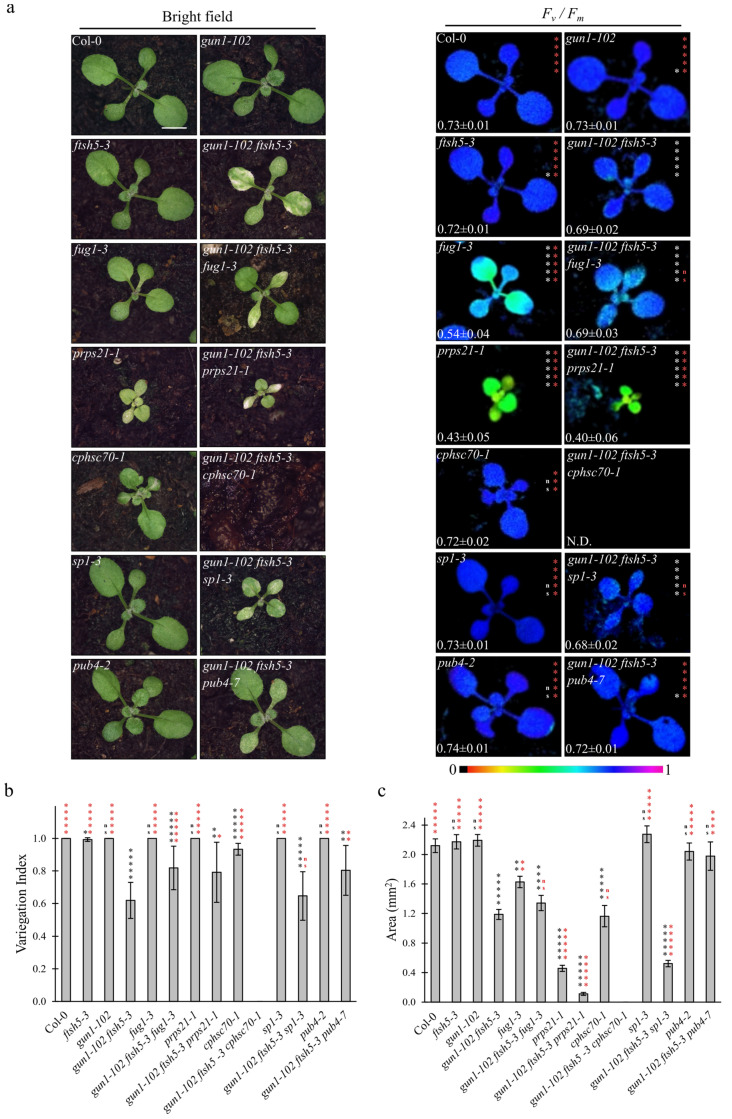
Visible phenotypic characteristics and photosynthetic performance of Arabidopsis Col-0 and mutant seedlings at two-leaves stage. (**a**) Visible phenotypes and maximum quantum yield of PSII (*F_v_/F_m_*) of Col-0, *gun1-102*, *ftsh5-3*, *gun1-102 ftsh5-3*, *fug1-3*, *gun1-102 ftsh5-3 fug1-3*, *prps21-1*, *gun1-102 ftsh5-3 prps21-1*, *cphsc70-1*, *gun1-102 ftsh5-3 cphsc70-1*, *sp1-3*, *gun1-102 ftsh5-3 sp1-3*, *pub4-2* and *gun1-102 ftsh5-3 pub4-7* true leaves at 12 days after sowing (DAS) grown on soil. Scale bar corresponds to 1 cm. *F_v_/F_m_* parameter was measured through the IMAGING PAM Fluorimeter (Walz) and reported in false colour (black equals to 0, violet to 1) and as average ± standard deviation of at least six independent measurements; N.D.: not detected. Asterisks indicate statistical significance with respect to Col-0 (white) or *gun1-102 ftsh5-3* (red) as evaluated by Student’s *t*-test and Welch correction (* *p* < 0.05; ** *p* < 0.01; *** *p* < 0.001; **** *p* < 0.0001; ***** *p* < 0.00001; ns, not significant). (**b**) Average Variegation Index (V.I.) calculated as ratio between green area and total area of the leaf of Col-0 and mutants grown on soil at 12 DAS. Error bars indicate standard deviations of at least six independent measurements. Asterisks indicate statistical significance with respect to Col-0 (black) or *gun1-102 ftsh5-3* (red) as evaluated by Student’s *t*-test and Welch correction (* *p* < 0.05; ** *p* < 0.01; *** *p* < 0.001; **** *p* < 0.0001; ***** *p* < 0.00001; ns: not significant). (**c**) Average area in mm^2^ of single first leaves of the indicated genotypes grown on soil at 12 DAS. Error bars indicate standard deviations of at least six independent measurements. Asterisks indicate statistical significance with respect to Col-0 (black) or *gun1-102 ftsh5-3* (red) as evaluated by Student’s *t*-test and Welch correction (* *p* < 0.05; ** *p* < 0.01; *** *p* < 0.001; **** *p* < 0.0001; ***** *p* < 0.00001; ns: not significant).

To dissect the molecular mechanisms responsible for the variegated phenotype observed in *gun1-102 ftsh5-3* cotyledons and true leaves, as a consequence of chloroplast protein homeostasis perturbation ([16]; Figure 1a and Figure 2a), we introgressed mutations in genes involved in: (i) plastid translation, *fug1-3* and *prps21-1* [32,33]; (ii) plastid protein folding/import, *cphsc70-1* [34,35]; (iii) cytosolic protein ubiquitination, *sp1-3* and *pub4-7* [22,24] (see Figure S1 and Table 1), with the aim of identifying Arabidopsis triple mutants able to suppress the *gun1-102 ftsh5-3* double mutant phenotype. The large majority of the mutations investigated are caused by T-DNA insertions in the transcribed regions of the genes of interest, resulting in knock-out alleles. The only exception is represented by *fug1-3* knock-down allele caused by a T-DNA insertion in the promoter region of *At1g17220* locus (see Figure S1). Moreover, since the *PUB4* and *GUN1* loci are located on adjacent regions of chromosome 2, the *PUB4* gene was silenced in the *gun1-102 ftsh5-3* genetic background by using the CRISPR-Cas9 gene editing strategy. In particular, the *pub4-7* allele is due to a frameshift mutation caused by the insertion of a Thymine in the fourth exon of the *At2g23140* gene, in position +1804 from the transcription starting site (see Figure S1).

The obtained triple mutants were analysed at 6 DAS (Figure 1) and 12 DAS (Figure 2) to evaluate the Variegation Index (V.I.; see also Materials and Methods) and the total organ area in cotyledons and the first true leaves (Figure 1b,c and Figure 2b,c). Furthermore, the functionality of chloroplasts was also assessed by determining their photosynthetic performance through the measurement of *F_v_/F_m_* (Maximum quantum yield of PSII) and Y_II_ (Effective quantum yield of PSII) parameters (Figure 1a and Figure 2a and Figure S2a), (b). Strikingly, the *gun1-102 ftsh5-3 pub4-7* triple mutant was the only one showing a marked improvement of all the considered parameters at 6 and 12 DAS.

The significant increase in V.I. in both cotyledons and leaves, as well as the higher values of both F_v_/F_m_ and Y_II_ parameters, together with the increased total area of both cotyledons and first true leaves (see Figure 1c and Figure 2c), indicated that the *pub4-7* allele in *gun1-102 ftsh5-3 pub4-7* seedlings was the only second-site mutation able to suppress the variegated phenotype of *gun1-102 ftsh5-3* cotyledons and leaves. Indeed, *gun1-102 ftsh5-3 prps21-1* seedlings showed an even exacerbated phenotype in terms of V.I. and photosynthetic performance at the cotyledon stage, while the *gun1-102 ftsh5-3 cphsc70-1* triple mutant exhibited seedling lethality. In the latter case, the few seedlings able to germinate showed reduced white cotyledons virtually devoid of chloroplasts, as their photosynthetic parameters were undetectable. Only in the case of *gun1-102 ftsh5-3 sp1-3* and *gun1-102 ftsh5-3 fug1-3* cotyledons was the non-additive effect observed in terms of variegation and photosynthetic parameters. However, the total area of cotyledons slightly increased in *gun1-102 ftsh5-3 fug1-3*. Conversely, introgression of *sp1-3* led to a small but significant reduction in cotyledon size while, at 12 DAS, the first true leaves of *gun1-102 ftsh5-3 sp1-3* plants were notably smaller than those of *gun1-102 ftsh5-3*. Interestingly, *gun1-102 ftsh5-3 prps21-1* and *gun1-102 ftsh5-3 fug1-3* true leaves showed a statistically significant increase in V.I. in comparison with the leaves of *gun1-102 ftsh5-3* double mutant, indicating that both the plastid protein synthesis rate and cytosolic protein ubiquitination could contribute to the onset of leaf variegation, upon perturbation of chloroplast protein homeostasis.

### 3.2. PUB4 E3 Ubiquitin Ligase Plays a Major Role in Chloroplast Degradation upon Alteration of Plastid Protein Homeostasis

**Figure 3:**
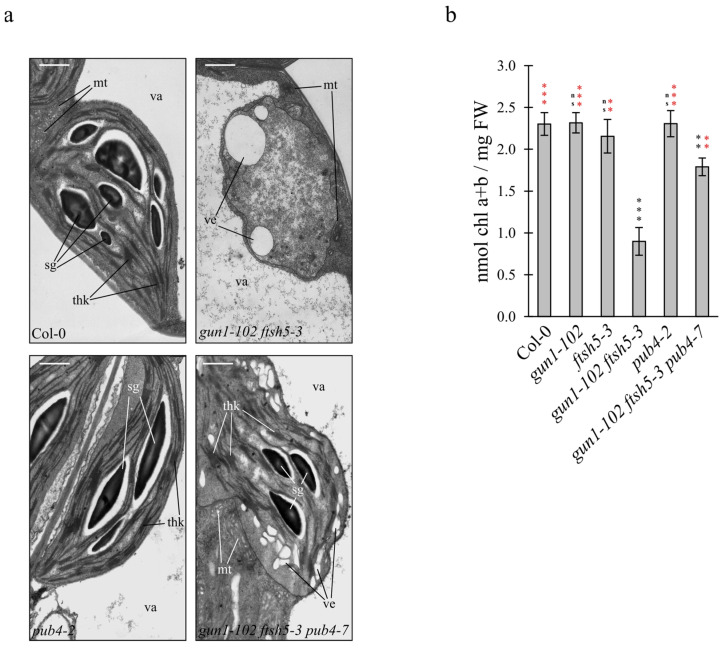
Chloroplast ultrastructure and chlorophyll content of Arabidopsis Col-0 and mutant cotyledons. (**a**) TEM micrographs of chloroplasts in mesophyll cells of Col-0, *gun1-102 ftsh5-3*, *pub4-2* and *gun1-102 ftsh5-3 pub4-7* cotyledons at 6 DAS. Ultrathin sections of cotyledons from Col-0 and mutant seedlings were stained with 2% uranyl acetate and lead citrate and examined by TEM. Scale bars correspond to 1 µm. The main cellular structures are indicated (sg: starch granule; thk: thylakoid membranes; ve: budding vesicles; mt: mitochondrion; va: vacuole). (**b**) Cotyledon chlorophyll content of the indicated Arabidopsis genotypes grown on soil at 6 DAS. The total chlorophyll content is normalised on the cotyledon fresh weight (nmol Chl a + b/mg FW). Asterisks indicate statistical significance with respect to Col-0 (black) or *gun1-102 ftsh5-3* (red) as evaluated by Student’s *t*-test and Welch correction (** *p* < 0.01; *** *p* < 0.001; ns: not significant).

To further investigate the role of PUB4 as a component of the chloroplast quality control machinery (see also [16]), thin sections of cotyledons from the Col-0, *gun1-102 ftsh5-3* double mutant, *pub4-2* T-DNA insertional mutant (Figure S1) and *gun1-102 ftsh5-3 pub4-7* triple mutant were analysed by Transmission Electron Microscope (TEM) (Figure 3a). Chloroplasts of Col-0 and *pub4-2* cotyledons appeared to be correctly shaped, with the proper thylakoid ultrastructure organization in grana stacks and stroma lamellae and abundant starch granules in the stroma, as in the case of *gun1-102* and *ftsh5-3* chloroplasts (Figure 3a; see also Tadini et al. [16]). In agreement with the V.I. values, several plastids devoid of thylakoid membranes and with large vesicles either inside the stroma (Figure 3a) or budding from the envelope, as also reported in Tadini et al. [16], were observed in *gun1-102 ftsh5-3* cotyledon cells, indicating advanced chloroplast degradation.

On the contrary, *gun1-102 ftsh5-3 pub4-7* cotyledons contained functional chloroplasts with severely altered shape, but that were still able to accumulate thylakoid membranes organized in grana and stroma lamellae and that were capable of performing photosynthesis, as proven by the accumulation of starch granules (Figure 3a). The suppression of *gun1-102 ftsh5-3* variegated phenotype, achieved by the introgression of *pub4-7* mutation, was also investigated by measuring the cotyledon chlorophyll content through a spectrophotometer-based assay (Figure 3b). Chlorophyll content was similar in Col-0, *gun1-102*, *ftsh5-3* and *pub4-2* cotyledons, while it was reduced to about 60% of control levels in *gun1-102 ftsh5-3* seedlings. In agreement with previous observations (Figure 1 and Figure 3a), *gun1-102 ftsh5-3 pub4-7* cotyledons showed almost double the amount of chlorophyll compared to the variegated *gun1-102 ftsh5-3* cotyledons.

### 3.3. The Absence of PUB4 E3 Ubiquitin Ligase Activity Increases Cytosolic Protein Folding Stress

**Figure 4:**
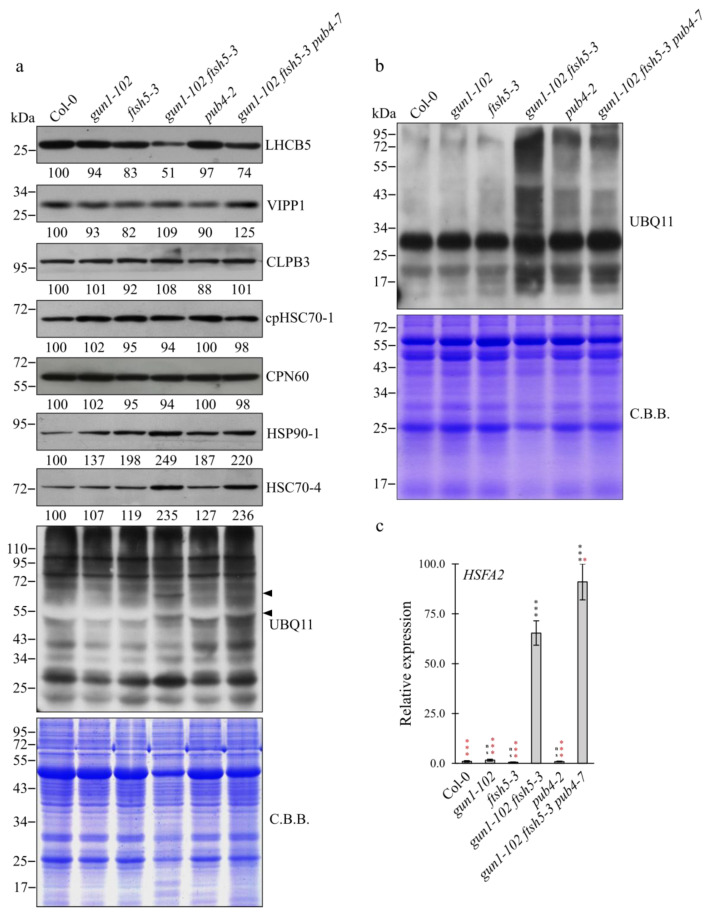
Protein and transcript accumulation in Arabidopsis Col-0 and mutant cotyledons. (**a**) Immuno-blot analyses of total protein extracts from cotyledons of Col-0, *gun1-102*, *ftsh5-3*, *gun1-102 ftsh5-3*, *pub4-2* and *gun1-102 ftsh5-3 pub4-7* grown on soil at 6 DAS. PVDF filters bearing fractionated total proteins were incubated with antibodies raised against LHCB5 antenna protein, VIPP1 plastid membrane chaperone, CLPB3 plastid unfoldase, cpHSC70-1 plastid chaperone, CPN60 plastid chaperonin, HSP90-1 cytosolic chaperone, HSC70-4 cytosolic chaperone and UBQ11 ubiquitin protein. Coomassie Brilliant Blue (CBB) stained SDS-PAGE is included as loading control. (**b**) Immunoblot analyses of chloroplast protein extracts from Col-0, *gun1-102*, *ftsh5-3*, *gun1-102 ftsh5-3*, *pub4-2* and *gun1-102 ftsh5-3 pub4-7* grown on soil at 6 DAS. PVDF filters bearing fractionated proteins were incubated with antibodies raised against UBQ11 ubiquitin protein. CBB-stained SDS-PAGE is included as loading control. (**c**) Relative expression level of *HSFA2* gene determined by qRT-PCR analyses of total RNA extracted from cotyledons of Col-0, *gun1-102*, *ftsh5-3*, *gun1-102 ftsh5-3*, *pub4-2* and *gun1-102 ftsh5-3 pub4-7* grown on soil at 6 DAS. Error bars indicate standard deviations of three replicates. Asterisks indicate statistical significance with respect to Col-0 (black) or *gun1-102 ftsh5-3* (red) as evaluated by Student’s *t*-test and Welch correction (* *p* < 0.05; *** *p* < 0.001; ns: not significant).

Immunoblot analyses of total protein extracts from Col-0, *gun1-102*, *ftsh5-3*, *pub4-2*, *gun1-102 ftsh5-3* and *gun1-102 ftsh5-3 pub4-7* cotyledons were performed to monitor the accumulation of components of the thylakoid electron transport chain, i.e., LHCB5, of thylakoid membrane biogenesis/maintenance, such as VIPP1 [37,38], of the plastid protein homeostasis machinery, such as CLPB3, cpHSC70-1 and CPN60 [34,39,40] and of the plastid pre-protein guidance complex and molecular markers of the cytosolic folding stress, as in the case of HSC70-4 and HSP90-1 cytosolic chaperones [17,26,41] (Figure 4a).

In agreement with previous observations, the higher level of LHCB5 in *gun1-102 ftsh5-3 pub4-7* cotyledons with respect to *gun1-102 ftsh5-3* further supported the ability of *pub4-7* mutation to suppress the variegated phenotype of *gun1-102 ftsh5-3* cotyledons. Furthermore, the accumulation of VIPP1 appeared comparable among all the genotypes except for *gun1-102 ftsh5-3 pub4-7*, which showed a slight increase in VIPP1 levels, while CLPB3, cpHSC70-1 and CPN60 resulted unchanged in all the genotypes. Interestingly, the accumulation of HSC70-4 and HSP90-1 cytosolic chaperones increased markedly in both the *gun1-102 ftsh5-3* double mutant and *gun1-102 ftsh5-3 pub4-7* triple mutant (Figure 4a) in line with what observed in *gun1* single mutant upon Lincomycin treatment [16,17], pointing to a relatively high cytosolic protein folding stress in both mutant backgrounds. Finally, the total protein ubiquitination level was assessed by a UBQ11 specific antibody. Overall, no evident difference in total signal intensity was observed between wild-type and the different mutant samples. However, the protein ubiquitination pattern in *gun1-102 ftsh5-3* cotyledons showed two relatively strong bands at around 70 and 55 kDa (see arrow heads in Figure 4a), not detectable in Col-0 and in the single mutant cotyledons. Strikingly, the band at 55 kDa was also visible in *gun1-102 ftsh5-3 pub4-7* cotyledons, while the signal at 70 kDa disappeared in the triple mutant genetic background (Figure 4a). The lack of important discrepancies in the ubiquitination levels among all of the genotypes, even in the absence of PUB4 E3 ubiquitin ligase enzyme, prompted us to investigate whether major differences could be observed at the chloroplast level, given the ability of PUB4 to promote chloroplast degradation upon alteration of plastid protein homeostasis (see above). To this aim, intact purified chloroplasts were isolated from cotyledons of Col-0 and mutant seedlings and chloroplast proteins probed with the UBQ11 specific antibody (see Figure 4b). While chloroplast ubiquitination levels of Col-0, *gun1-102* and *ftsh5-3* chloroplasts were rather similar and most of the ubiquitination signal was concentrated in a single band migrating at around 30 kDa, the plastid ubiquitination levels increased largely in the *gun1-102 ftsh5-3* genetic background, where signals of ubiquitinated proteins could be also detected at molecular weights different from the 30 kDa main band (Figure 4b). Intriguingly, a large part of the increased ubiquitination signals disappeared upon depletion of PUB4 activity in *gun1-102 ftsh5-3 pub4-7* chloroplasts (Figure 4b). In addition, the relative expression of *HSFA2* transcription factor, a key regulator of the chloroplast-related nuclear stress response [42,43], was assessed via qRT-PCR (Figure 4c). Consistently with the increase in cytosolic chaperone accumulation (see Figure 4a), *HSFA2* transcripts were strongly up-regulated in *gun1-102 ftsh5-3* and, to an even larger extent, in *gun1-102 ftsh5-3 pub4-7* seedlings.

## 4. Discussion

Variegated mutants represent an important genetic tool to investigate chloroplast biogenesis and to dissect the functional interactions of different pathways involved in plastid differentiation, chloroplast protein homeostasis, chloroplast quality control and degradation. In this manuscript, we studied the GUN1-mediated plastid development and degradation, in the context of altered plastid protein homeostasis, as shown by the variegated phenotype of *gun1-102 ftsh5-3* seedlings.

**Figure 5:**
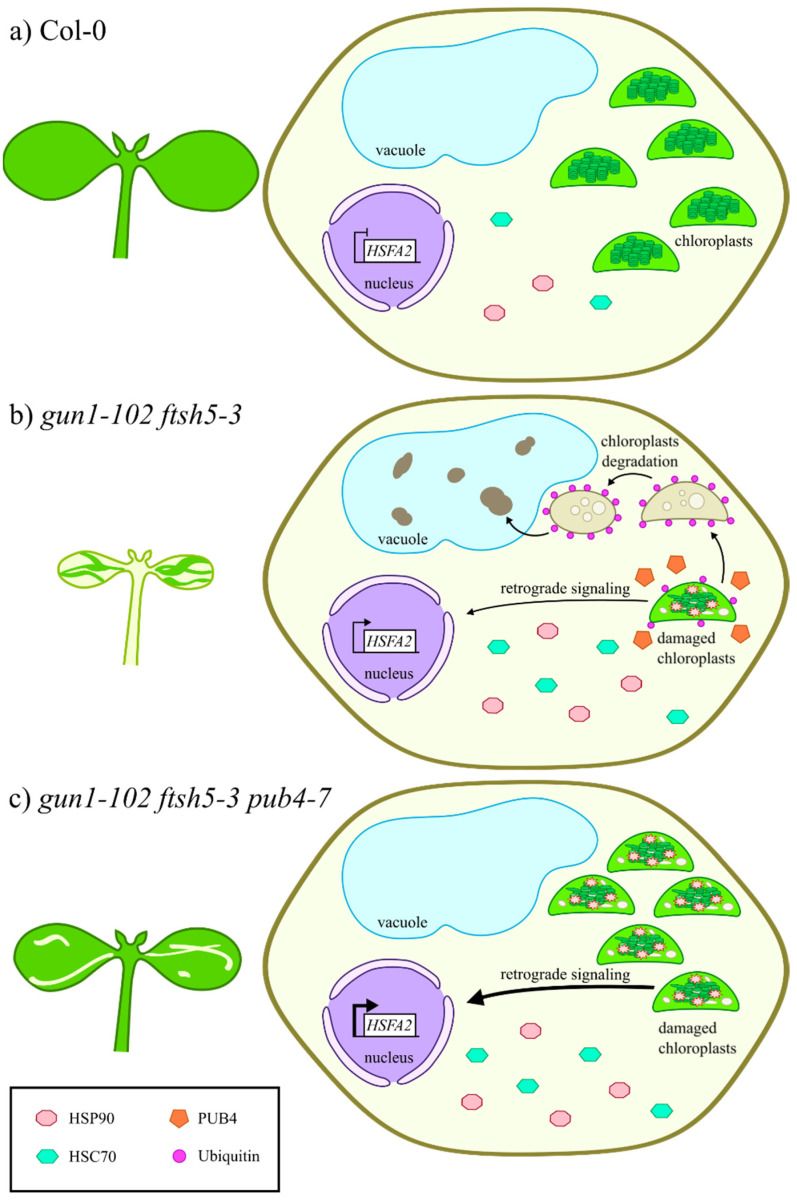
Schematic overview representing the *pub4*-mediated suppression of variegation. (**a**) In Col-0 seedlings, cells display functional chloroplasts, physiological cytosolic folding stress and clear vacuoles. (**b**) On the contrary, chloroplasts in advanced degradation stages and vacuoles filled with electron-dense material are visible in *gun1-102 ftsh5-3* variegated cotyledons. The electron dense material inside the vacuole might derive from plastid degradation. The expression of the stress-responsive *HSFA2* nuclear gene is strongly up-regulated, in response to signals from chloroplasts with altered protein homeostasis. Furthermore, the accumulation of cytosolic chaperones, namely HSP90 and HSC70, is increased, while the removal of damaged chloroplasts is favoured by PUB4 ubiquitin ligase. (**c**) The absence of PUB4 in the *gun1-102 ftsh5-3 pub4-7* genetic background largely prevents the degradation of damaged chloroplasts, resulting in the suppression of variegation in both cotyledons and true leaves. Nevertheless, damaged chloroplasts, still functioning, trigger stress-related retrograde signals and lead to even higher *HSFA2* expression levels.

As the *gun1-102 ftsh5-3* double mutant possesses chloroplasts prone to suffer terminal damages, the recovery of *gun1-102 ftsh5-3 pub4-7* phenotype observed in cotyledons and true leaves (Figure 1 and Figure 2) suggests that PUB4-mediated ubiquitination is the preferential pathway for degrading and recycling damaged chloroplasts upon alteration of plastid protein homeostasis (Figure 5). These observations are in agreement with previous studies, in which ROS-damaged chloroplasts in Arabidopsis plants lacking the plastid ferrochelatase 2 (*fc2-1*) exhibited a PUB4-dependent increase in poly-ubiquitinated proteins [24]. The altered shape of chloroplasts observed in *gun1-102 ftsh5-3 pub4-7* cotyledons (Figure 3a) is compatible with the early steps of the degradation process, not effectively achieved or delayed due to the absence of PUB4 protein activity, as previously reported [24]. In line with this, the up-regulation of the stress-responsive gene *HSFA2*, here used as molecular marker of damaged chloroplasts and the related nuclear stress response [42,43], observed in *gun1-102 ftsh5-3*, was comparable or even higher in *gun1-102 ftsh5-3 pub4-7*, proving that *pub4* mutation does not suppress the damage itself (Figure 4). It is tempting to explain the higher *HSFA2* expression as a consequence of the increased number of chloroplasts in cells that, although defective, are still performing photosynthesis and, consequently, generating ROS and other stress-related signals. Worth noticing, *HSFA2* expression is triggered by a GUN1-indepentent retrograde signalling pathway, as previously observed [43]. Furthermore, the introgression of *pub4-7* mutation does not suppress the increased cytosolic folding stress observed in *gun1-102 ftsh5-3* cotyledons (Figure 4), and previously described in *gun1* seedlings upon lincomycin treatment [16,17] as proven by the over-accumulation of the cytosolic chaperones HSC70-4 and HSP90-1 and the increased total protein ubiquitination level. Noteworthy is that the comparison between *gun1-102 ftsh5-3* and *gun1-102 ftsh5-3 pub4-7* ubiquitination profiles, performed, for example, by mass spectrometry analyses of ubiquitin-enriched protein extracts, seems to be an optimal strategy to identify those chloroplast outer envelope proteins that are modified by the PUB4 ubiquitin ligase.

On the contrary, the *sp1-3* mutation, which abolishes the activity of a transmembrane ubiquitin ligase responsible for controlling TOC protein complex composition and activity, does not suppress variegation in *gun1-102 ftsh5-3* cotyledons and leaves (Figure 1 and Figure 2). SP1 activity has been recognized as essential for chloroplast biogenesis in etiolated seedlings and to play a role in the tolerance of abiotic stresses, by modulating the import rate of photosynthetic proteins, and, therefore, to prevent ROS accumulation [22,25,26]. In light of this, the *gun1-102 ftsh5-3 sp1-3* triple mutant is devoid of an additional level of chloroplast quality control mechanism that results in an overall decrease in plant growth with respect to *gun1-102 ftsh5-3* (Figure 1 and Figure 2). Taken together, these observations corroborate the model in which ubiquitination of specific chloroplast proteins triggers different mechanisms of stress tolerance, in cotyledons and leaves, not only in response to abiotic stresses but also as adaptation to genetic defects.

The introgression of *cphsc70-1* mutation, impaired in plastid protein import and protein folding [34,35], into the *gun1-102 ftsh5-3* genetic background led to a complete seedling lethal phenotype. This is in agreement with previous results, as *GUN1* was shown to have functional interactions with *FTSH5* and *cpHSC70-1* loci [16,17]. The fact that *ftsh5-3 cphsc70-1* double mutant displays no additive phenotype compared to *cphsc70-1* (Figure S3), while the *gun1-102 ftsh5-3 cphsc70-1* shows seedling-lethality (Figure 1), suggests that plastid protein import, protein folding and plastid proteolysis act synergistically for achieving the correct chloroplast development and GUN1 is essential for such orchestration.

At last, in line with previous observations [10,32,33], the suppression of variegated phenotype was effectively achieved in *gun1-102 ftsh5-3* true leaves by reducing the plastid translation rate, driven by the introgression of either *fug1-3* or *prps21-1* mutated alleles. Strikingly, this compensatory effect failed to occur in cotyledons, proving further that the pathways that underlie chloroplast biogenesis in cotyledons and leaves are characterized by substantial differences.

## Figures and Tables

**Table 1:** List of mutant lines analysed in this study. Gene names, AGI accession numbers, subcellular localization of the gene products, functions and references where mutant alleles have been described are included.

Gene Name	AGI Code	Subcellular Localization	Allele	Function
*GUN1*	At2g31400	chloroplast stroma	*gun1-102* [19]	PPR protein involved in chloroplast-to-nucleus communication and maintenance of plastid protein homeostasis [16,17,18,20].
*FTSH5*	At5g42270	chloroplast thylakoids	*ftsh5-3* [19]	Transmembrane protease involved in thylakoid biogenesis and PSII maintenance by removal of damaged D1 subunit [6].
*FUG1*	At1g17220	chloroplast stroma	*fug1-3* [32]	Initiation factor essential for plastid protein translation [32].
*PRPS21*	At3g27160	chloroplast stroma	*prps21-1* [19]	Structural component of the 30S plastid ribosome subunit [36].
*cpHSC70-1*	At4g24280	chloroplast stroma	*cphsc70-1* [34]	Plastid chaperone involved in protein import and folding processes [34].
*SP1*	At1g63900	cytosolic side of the plastid outer envelope	*sp1-3* [22]	E3 ubiquitin ligase involved in the regulation of plastid protein import and component of CHLORAD, i.e., ubiquitination and retro-translocation of outer membrane proteins for proteasome-dependent degradation [22].
*PUB4*	At2g23140	cytosol	*pub4-2* [24] *pub4-7* [This work]	E3 ubiquitin ligase associated with the selective degradation of ROS-damaged chloroplasts [24].

## Supplementary Materials

The following are available online at https://www.mdpi.com/article/10.3390/genes12091387/s1, Figure S1: Graphical representations of the loci mentioned in this study, Figure S2: Photosynthetic performance of Arabidopsis Col-0 and mutant seedlings, Figure S3: Visible phenotypic characteristics and Variegation Index of Arabidopsis Col-0 and mutant seedlings at cotyledon stage, Table S1: Sequences of oligonucleotides employed for the molecular characterization of mutant lines.
