# Supplementary material for: The PUB4 E3 Ubiquitin Ligase Is Responsible for the Variegated Phenotype Observed upon Alteration of Chloroplast Protein Homeostasis in Arabidopsis Cotyledons
Source: Genes (Basel). 2021 Sep 6;12(9):1387. doi: 10.3390/genes12091387 (PMC8464772; doi:10.3390/genes12091387)
Supplement: Supplementary file 1 [file genes-12-01387-s001.zip › Manuscript_supplementary.pdf]

Figure S1 Graphical representations of the loci mentioned in this study. T-DNA tagging and CRISPR-Cas9 gene editing are indicated, together with their positions, calculated from the transcriptional start site, and the name of the different vectors used for plant transformation. The positions of translation start and stop codons are also indicated for each gene. RB, right border; LB, left border of the T-DNA insertion, which is not drawn to scale. The names of the different T-DNA insertional alleles are reported above the T-DNA description, with the exception of *pub4-7* mutant allele, where the insertion of a Thymine (T) in the fourth exon, obtained by CRISPR-Cas9 gene editing strategy, is indicated.

Figure S2 Photosynthetic performance of Arabidopsis Col-0 and mutant seedlings. **a)** Average effective quantum yield of PSII (Y_{II}) of the indicated genotypes grown on soil at 6 DAS measured through the IMAGING PAM Fluorimeter (Walz). Error bars indicate standard deviations of at least six independent measurements. Asterisks indicate statistical significance with respect to Col-0 (black) or *gun1-102 ftsh5-3* (red) as evaluated by Student's t-test and Welch correction (*P < 0,05; **P < 0,01; ***P < 0,001; ****P < 0,0001; *****P < 0,00001; ns: not significant). **b)** Average effective quantum yield of PSII (Y_{II}) of the indicated genotypes grown on soil at 12 DAS measured through the IMAGING PAM Fluorimeter (Walz). Error bars indicate standard deviations of at least six independent measurements. Asterisks indicate statistical significance with respect to Col-0 (black) or *gun1-102 ftsh5-3* (red) as evaluated by Student's t-test and Welch correction (*P < 0,05; **P < 0,01; ***P < 0,001; ****P < 0,0001; *****P < 0,00001; ns: not significant).

Figure S3 Visible phenotypic characteristics and Variegation Index of Arabidopsis Col-0 and mutant seedlings at cotyledon stage. **a**) Visible phenotypes of *ftsh5-3, cphsc70-1* and *ftsh5-3 cphsc70-1* grown on soil at 6 DAS. Scale bar corresponds to 1 mm. **b**) Average Variegation Index (V.I.) calculated as ratio between the green area over the total area of the cotyledon of Col-0 and mutant seedlings grown on soil at 6 DAS. Error bars indicate standard deviations of at least six independent measurements. The comparison between *cphsc70-1* and *ftsh5-3 cphsc70-1* resulted in no statistically significant difference (ns: not significant).

Table S1 Sequences of oligonucleotides employed for the molecular characterization of mutant lines.
