# Supplementary figures and images for: The PUB4 E3 Ubiquitin Ligase Is Responsible for the Variegated Phenotype Observed upon Alteration of Chloroplast Protein Homeostasis in Arabidopsis Cotyledons
Source: Genes (Basel). 2021 Sep 6;12(9):1387. doi: 10.3390/genes12091387 (PMC8464772; doi:10.3390/genes12091387)

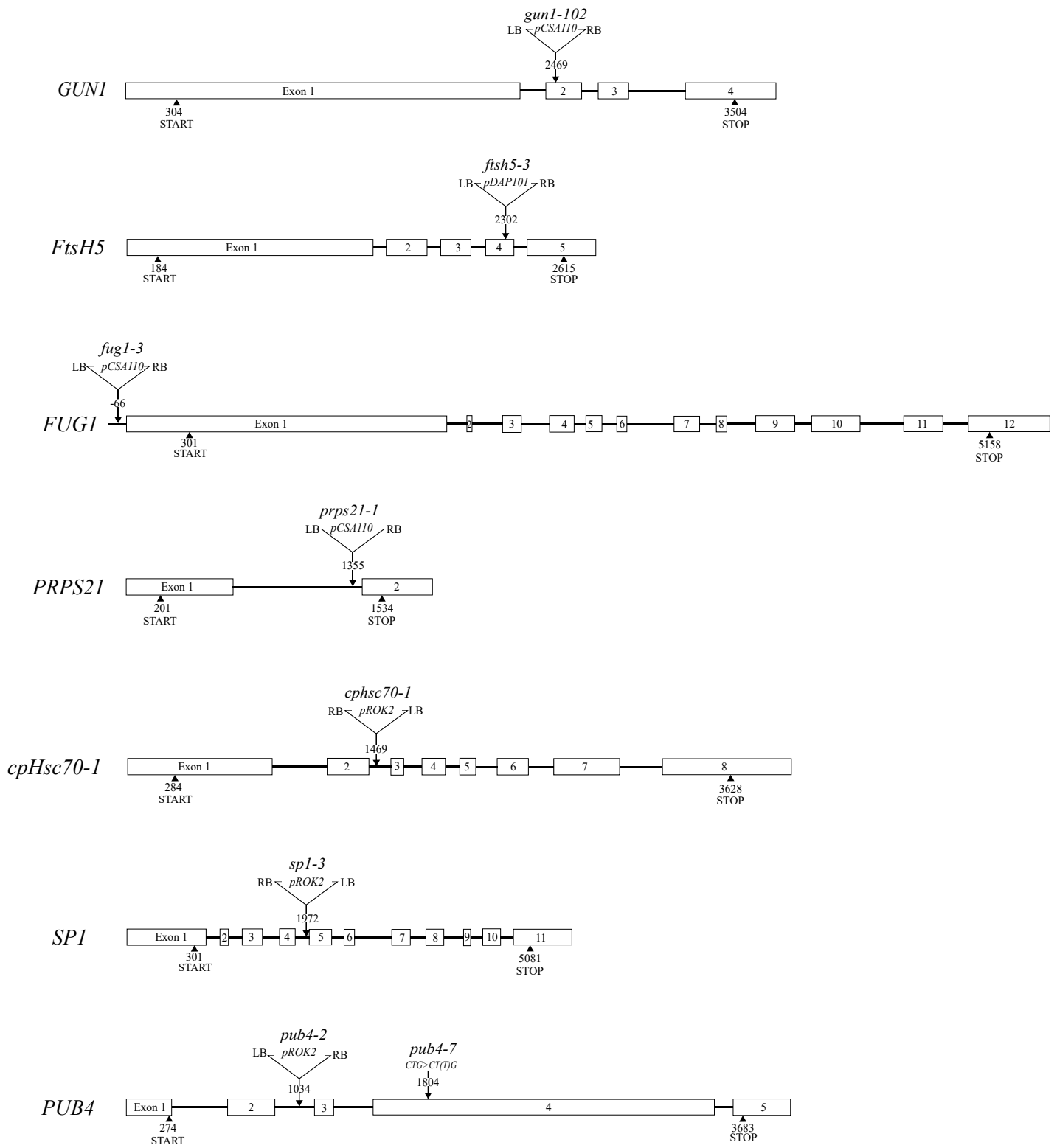

Supplement: Supplementary file 1 [file genes-12-01387-s001.zip › FigS1.pdf]

a

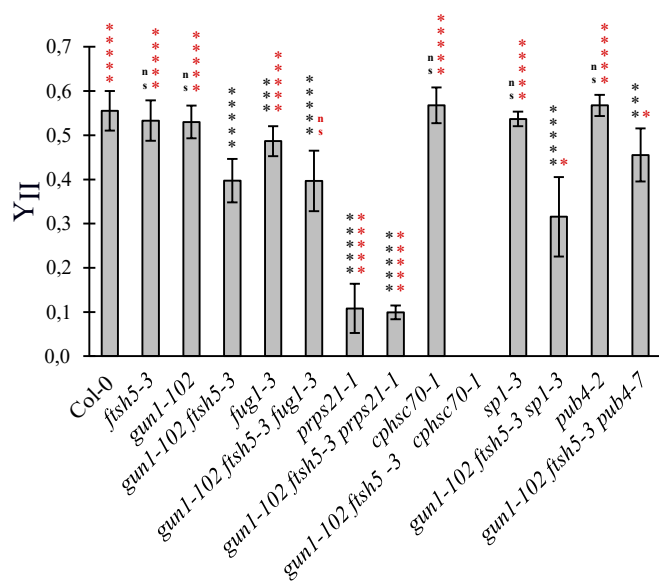

b

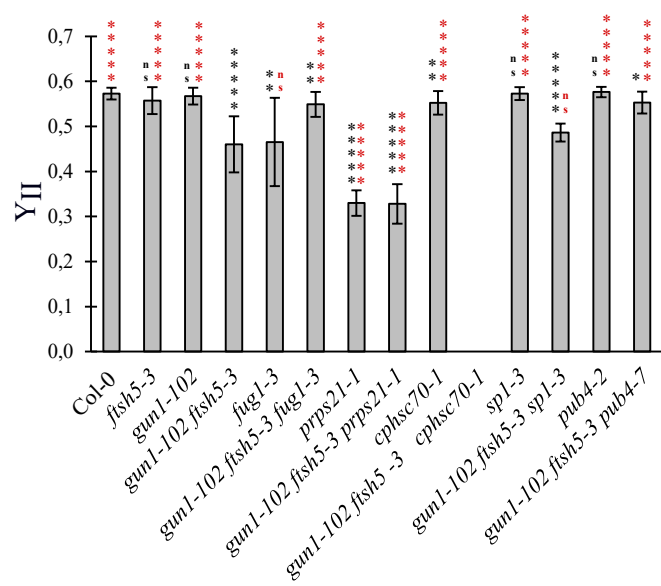

Supplement: Supplementary file 1 [file genes-12-01387-s001.zip › FigS2.pdf]

a

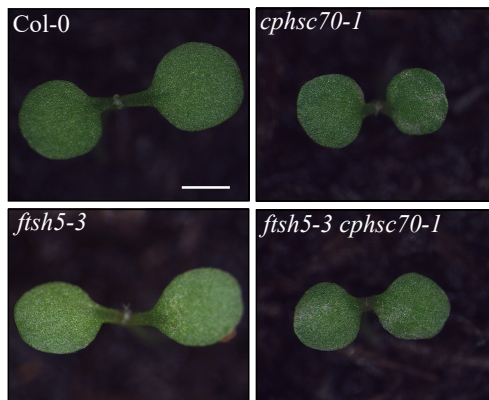

b

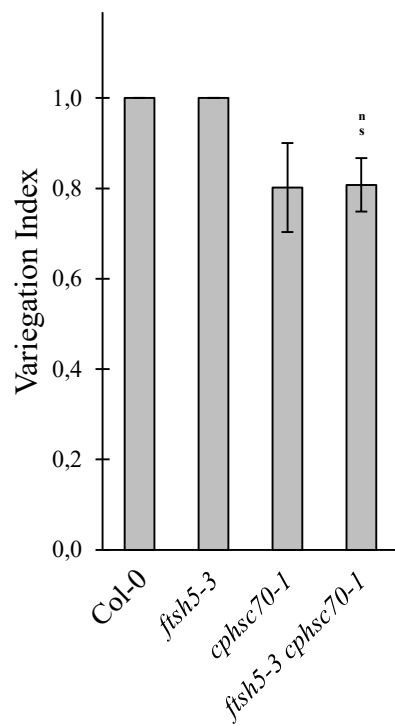

Supplement: Supplementary file 1 [file genes-12-01387-s001.zip › FigS3.pdf]
